# The Effects of Sindbis Viral Vectors on Neuronal Function

**DOI:** 10.3389/fncel.2019.00362

**Published:** 2019-08-08

**Authors:** Seçil Uyaniker, Sophie J. F. van der Spek, Niels R. Reinders, Hui Xiong, Ka Wan Li, Koen Bossers, August B. Smit, Joost Verhaagen, Helmut W. Kessels

**Affiliations:** ^1^Laboratory for Neuroregeneration, The Netherlands Institute for Neuroscience, Royal Netherlands Academy of Arts and Sciences, Amsterdam, Netherlands; ^2^Department of Molecular and Cellular Neurobiology, Center for Neurogenomics and Cognitive Research, Amsterdam Neuroscience, VU University Amsterdam, Amsterdam, Netherlands; ^3^Department of Cellular and Computational Neuroscience, Swammerdam Institute for Life Sciences, Amsterdam Neuroscience, University of Amsterdam, Amsterdam, Netherlands

**Keywords:** Sindbis viral vector, hippocampus, transcriptomics, proteomics, electrophysiology

## Abstract

Viral vectors are attractive tools to express genes in neurons. Transduction of neurons with a recombinant, replication-deficient Sindbis viral vector is a method of choice for studying the effects of short-term protein overexpression on neuronal function. However, to which extent Sindbis by itself may affect neurons is not fully understood. We assessed effects of neuronal transduction with a Sindbis viral vector on the transcriptome and proteome in organotypic hippocampal slice cultures, and analyzed the electrophysiological properties of individual CA1 neurons, at 24 h and 72 h after viral vector injection. Whereas Sindbis caused substantial gene expression alterations, changes at the protein level were less pronounced. Alterations in transcriptome and proteome were predominantly limited to proteins involved in mediating anti-viral innate immune responses. Sindbis transduction did not affect the intrinsic electrophysiological properties of individual neurons: the membrane potential and neuronal excitability were similar between transduced and non-transduced CA1 neurons up to 72 h after Sindbis injection. Synaptic currents also remained unchanged upon Sindbis transduction, unless slices were massively infected for 72 h. We conclude that Sindbis viral vectors at low transduction rates are suitable for studying short-term effects of a protein of interest on electrophysiological properties of neurons, but not for studies on the regulation of gene expression.

## Introduction

Viral vector mediated gene transfer is a commonly used approach in neuroscience to deliver genetic material into neurons. An effective gene delivery vehicle for neuronal cells is a recombinant Sindbis viral vector (Schlesinger, 1993; Malenka and Marie, 2006). Sindbis is an *Alphavirus* with a positive sense single-stranded RNA genome that allows a relatively large gene packaging capacity (up to 6.5 kb) for proteins expressed under the control of a subgenomic RNA promoter. Recombinant, replication-deficient Sindbis virus-based vectors efficiently transduce neuronal cells (Gwag et al., 1998; Ehrengruber et al., 1999) and have been successfully used to express proteins of interest in neurons from dissociated cultures, organotypic slices and *in vivo* in order to study the cellular localization and function of these proteins (Maletic-Savatic et al., 1999; Osten et al., 2000; D’Apuzzo et al., 2001; Marie et al., 2005; Wang et al., 2011; Kebschull et al., 2016; Knafo et al., 2016; Reinders et al., 2016).

Sindbis viral vectors induce high levels of recombinant gene expression with a rapid onset: detectable levels of protein expression can be reached within 6 to 12 h after viral transduction (Gwag et al., 1998; Osten et al., 2000; D’Apuzzo et al., 2001). The levels of overexpression that can be achieved are substantial; for instance, Sindbis-mediated expression of AMPA-receptor subunits were approximately 5- to 10-fold increased relative to endogenously expressed AMPA-receptor subunits (Kessels et al., 2009). The fast induction and robust expression levels make Sindbis viral vectors highly suitable for studying effects of acute overexpression of a protein of interest on cell function, thus minimizing the risk of compensatory responses to the manipulation.

A concern for using the Sindbis virus-based expression system is its potential cytotoxicity. Although Sindbis viral vectors are less toxic to the host when the viral structural protein genes are deleted and only the gene of interest is expressed upon transduction, it can still cause shut-down of endogenous protein production within hours after transduction of heterologous cell lines (Bredenbeek et al., 1993; Frolov and Schlesinger, 1994). Possibly as a consequence of overwhelming the protein translation machinery, cytopathic effects begin to occur 30 to 48 h post-transduction (Bredenbeek et al., 1993; Frolov and Schlesinger, 1994). Post-mitotic neurons appear to be more tolerant toward recombinant Sindbis transduction: based on morphological and electrophysiological properties, hippocampal neurons transduced by Sindbis vectors remain viable for at least 48 h post-transduction (Ehrengruber et al., 1999; Maletic-Savatic et al., 1999; Marie et al., 2005; Kessels et al., 2009). However, the time course of potential disruptive events after transduction of neurons is not known. To obtain a complete picture of the state of a Sindbis transduced neuron, we set out to study the effects of Sindbis-mediated eGFP expression on the transcriptome, proteome, and electrophysiological properties of organotypic hippocampal slices at both 24 and 72 h post-transduction.

## Materials and Methods

### Preparation of Organotypic Hippocampal Slices

All protocols were approved by the Animal Welfare Authority at the Dutch Central Committee for Animal Experimentation (NVWA). All experimental procedures were approved by the Institutional Animal Care and Use Committee of the Royal Netherlands Academy of Arts and Sciences (KNAW). C57BL/6 mice were used in this study. 400 μm thick organotypic slices of the hippocampus were prepared from postnatal day 6–8 C57BL/6 mouse pups as described previously (Stoppini et al., 1991). After 7–12 days in culture for electrophysiology or after 8–9 days in culture for transcriptome and proteome analysis, Sindbis viral vector or PBS were injected into slices using glass pipets attached to a Picospritzer (General Valve, Fairfield, NJ, United States).

### Viral Vectors and Preparation

pSinRep5-eGFP expression plasmid was used, and infective Sindbis pseudovirions were produced using the helper vector pDH-BB according to the manufacturer’s protocol (Invitrogen BV). In short: RNA was produced from pSinRep5-eGFP and pDH-BB plasmids using mMESSAGE mMACHINE T7 Transcription Kit (Thermo Fisher Scientific). RNA was transfected into BHK-21 cell line by electroporation. After 48 h, Sindbis viral particles were isolated from BHK-21 supernatant through ultracentrifugation, resuspended in phosphate buffered saline (PBS) and stored at *−*80°C.

### Microarray Analysis

Three organotypic slices were pooled and total RNA was isolated using RNeasy Mini Kit (QiaGen, Valencia, CA, United States) according to manufacturer’s instructions. RNA concentration and purity were determined using a NanoDrop ND-1000 spectrophotometer (NanoDrop Technologies, Wilmington, DE, United States). RNA integrity (average RNA integrity number 9.1, SEM 0.14, range 7.5–10) was determined using the Agilent 2100 Bioanalyzer (Agilent Technologies, Palo Alto, CA, United States). Sample labeling and microarray hybridization were performed using Agilent 44K V2 Mouse Genome arrays according to manufacturer’s instructions (Agilent Technologies, Part Number G4846A). Briefly, 60 ng RNA from each individual sample was used to simultaneously amplify sample material and synthesize cRNA that is fluorescently labeled with either Cy3-CTP or Cy5-CTP (Low Input Quick Amp Labeling Kit, Two-Color, Agilent Technologies.) Prior to hybridization, 825 ng of each Cy3-CTP- and Cy5-CTP-labeled cRNA were mixed. Specifically, each hybridization consisted of two individual samples from the same mouse, one transduced with Sindbis and one sham-treated and collected at the same time point post-injection. In the mixed samples RNA was fragmented for 30 min at 60°C in 1x Fragmentation Buffer (Agilent Technologies). The fragmented RNA samples were hybridized to a microarray by incubating for 17h at 60°C in 1x Hi-RPM-Hybridization Buffer (Agilent Technologies) in a rotating hybridization chamber. After hybridization, the arrays were washed 6 times for 1 min each in saline-sodium phosphate–EDTA (SSPE)/0.005% N-Lauroylsarcosine (Sigma-Aldrich, St Louis, MO, United States), 1 min in 37°C 0.06x SSPE/0.005% N-Lauroylsarcosine and 30 s in acetonitrile (Sigma-Aldrich) at room temperature, then dried by nitrogen flow. Microarrays were scanned using an Agilent DNA Microarray Scanner at 5 mm resolution and 100% Photomultiplier tube setting. Microarray scans were quantified using Agilent Feature Extraction software (version 8.5.1). Raw expression data were imported into the R statistical processing environment using the LIMMA package in Bioconductor^1^. All features for which one or more foreground measurements were flagged as saturated or as non-uniformity outlier by the feature extraction software, were excluded from further analysis. As overall background levels were very low, no background correction was performed. The intensity distributions within and between arrays were normalized using the “quantile” algorithm in LIMMA. The log2-transformed intensity measurements per sample were used in all following analyses. To detect genes that are significantly up- or down-regulated upon Sindbis transduction, student’s *t*-test was used. Raw *P*-values were corrected for multiple testing using the Benjamini-Hochberg algorithm. For genes with FDR corrected *P*-values < 0.05 and fold changes (2, a gene ontology over-representation analysis was performed using the PANTHER overrepresentation test (PANTHER version 13.1) PANTHER Pathways, Panther GO Slim Biological Process, PANTHER GO Slim Molecular Function and PANTHER GO Slim Cellular Component annotation datasets. Bonferroni correction was used for multiple testing. Gene Ontology classes with a corrected P-value of <0.05 were considered significant. Micro-array data are available upon request.

### Proteomics Analysis

Snap-frozen organotypic slices (3 per group) were homogenized in ice-cold homogenization buffer (0.32 M Sucrose, 5 mM HEPES, pH 7.4) containing “cOmplete” protease inhibitor cocktail (Roche Applied Science, Indianapolis, IN, United States) with a glass hand homogenizer (12 strokes). Protein concentration was determined using a Bradford Assay. Subsequently SDS loading buffer was added to the samples. Sample complexity was reduced by separating 30 μg of protein per sample on molecular weight using a 10% SDS polyacrylamide gel. The gel was fixed overnight in fixation buffer (50% Ethanol and 28% Phosphoric Acid), washed three times in water and stained with Colloidal Coomassie Blue. Each sample was cut into two slices, the gel pieces were destained using 50mM NH_4_HCO_3_ and acetonitrile, and the proteins were digested with trypsin (sequence grade; Promega, Madison, United States) in a MultiScreen- HV 96 well plate (Millipore) overnight at 37°C. Finally, the peptides were extracted from the gel pieces using 0.1% Trifluoroacetic acid in 50% acetonitrile, and 0.1% Trifluoroacetic acid in 80% acetonitrile. The peptides were dried in a SpeedVac and stored at *−*20°C until further use. For HPLC-ESI MS/MS analysis, the TripleTOF 5600 + MS was coupled to an Ultimate 3000 LC system (Dionex). The samples were re-dissolved in mobile phase A (2% acetonitrile and 0.1% fluoroacetic acid), then loaded and trapped on a 5 mm Pepmap 100 C18 column (300 μm id, 5 μm particle size, Dionex). Next, peptides were fractionated on a Alltima C18 homemade column (300 μm id, 3 μm particle size), using a linear gradient of increasing concentration of mobile phase B (99.9% acetonitrile and 0.1% fluoroacetic acid) from 5% to 22% in 88 min, to 25% at 98 min, 40% at 108 min, and to 95% at 110 min. After 8 min. the column was back equilibrated to the initial condition of 5% acetonitrile for 10 min. Peptides were electrosprayed into the mass spectrometer using an ion spray voltage of 2.5 kV, Gas1 and Gas2 at 15 p.s.i., curtain gas at 25 p.s.i., and an interface heater temperature of 150°C. The MS survey scan ranged from m/z 350–1250 acquired for 200 ms. The top 20 precursor ions were selected for 100 ms per MS/MS acquisition, with a threshold of 100 counts and an exclusion window of 16s. Rolling CID function was activated, with an energy spread of 15 eV, and the subsequent MS/MS scan ranged from m/z 200–1800. Finally, the MS/MS spectra were searched against the Mouse (UP000000589_10090 and UP000000589_10090_additional) and Sindbis (UP0000006710_11034) database using the MaxQuant software (version 1.5.2.8). The search parameters were set to trypsin digestion, the rest was kept at default. The mass spectrometry proteomics data have been deposited to the ProteomeXchange Consortium via the PRIDE (Perez-Riverol et al., 2016, 2019; Deutsch et al., 2017) partner repository with the dataset identifier PXD013634.

### Electrophysiology

Organotypic hippocampal slices were perfused with ACSF (in mM: 118 NaCl, 2.5 KCl, 26 NaHCO_3_, 1 NaH_2_PO_4_, 4 MgCl_2_, 4 CaCl_2_, and 20 glucose) gassed with 95% O_2_/5% CO_2_. Whole-cell voltage-clamp recordings were made with 3 to 5 MΩ pipettes, and recordings were used when *R*_*access*_ < 20 MΩ, and *R*_*input*_ > 10 × *R*_*access*_. For mEPSC recordings, an internal solution was used containing (in mM) 115 CsMeSO_3_, 20 CsCl, 10 HEPES, 2.5 MgCl_2_, 4 Na_2_-ATP, 0.4 Na-GTP, 10 Na-Phosphocreatine, and 0.6 EGTA. Miniature EPSCs were recorded clamping at −60 mV with 1 μM TTX and 50 μM picrotoxin added to the bath, and were analyzed with the Mini Analysis program (Synaptosoft) with a minimum amplitude threshold of 5 pA. Neuronal excitabilities were recorded with internal solution containing (in mM) 130 K-gluconate, 20 KCl, 4 Mg-ATP, 0.3 Na-GTP, 10 HEPES, and 10 Na2-phosphocreatine. Current step recordings were analyzed with Clampex 10.7 (Molecular Devices). The average sag ratio was calculated from the *−*100 pA current injection as (1−ΔV_*ss*_/ΔVmin) × 100%, where ΔVss = Vrest−Vsteady state and ΔVmin = Vrest−Vminimum. All data were acquired using a Multiclamp 700B amplifier (Molecular Devices).

## Results

### Effects of Sindbis-GFP Transduction on the Transcriptome

**FIGURE 1:**
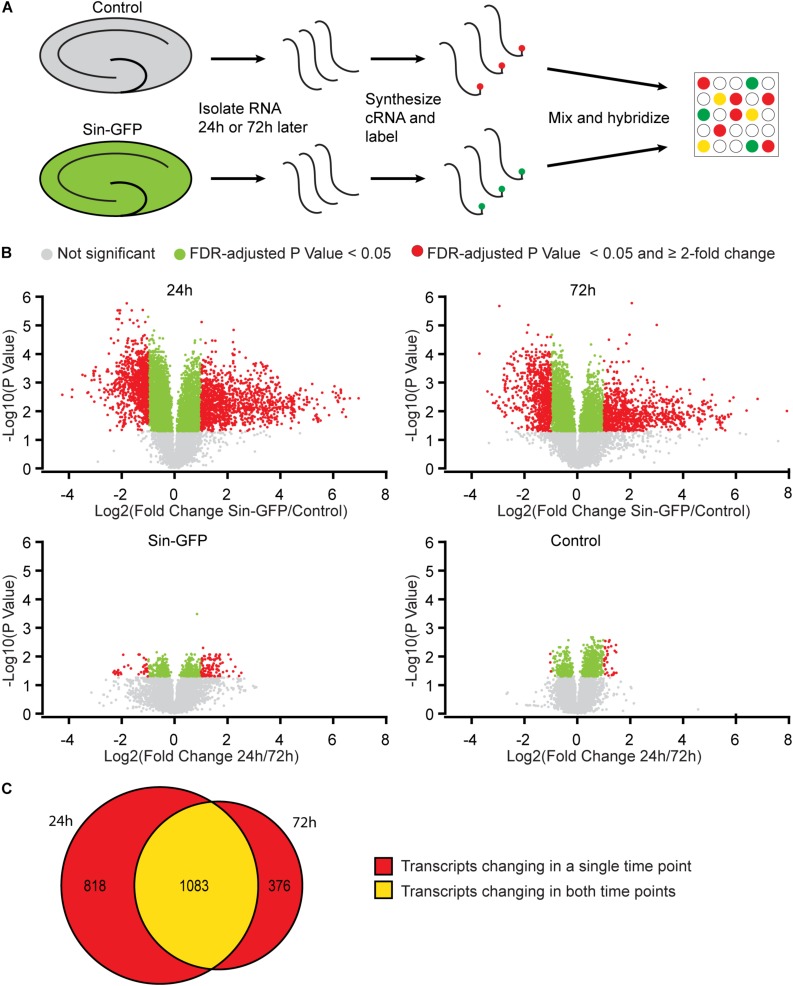
Gene expression changes in mouse hippocampal slices caused by Sindbis transduction. **(A)** Schematic workflow (see methods): Organotypic hippocampal slices were injected with PBS containing Sindbis-eGFP (*n* = 6) or with PBS (*n* = 6) as a control, and 24 or 72 h after injection RNA was isolated, labeled and hybridized to a mouse gene expression microarray. **(B)** Comparisons in gene expression between Sindbis-GFP and control samples at 24 h (upper left panel) and 72 h (upper right panel); and comparisons between 24 and 72h for Sindbis-GFP treated samples (lower left panel) and for control treated samples (lower right panel). Volcano plots show fold change in gene expression (Log2) against Benjamini-Hochberg corrected *p*-value (–Log10) for individual features on the microarray. Transcripts that do not pass the significance threshold (*p* < 0.05) are shown in grey, those that changed significantly <2-fold are shown in green, and those that changed significantly ≥2-fold are shown in red. **(C)** Venn diagram showing the overlap between the transcripts that change levels more than 2-fold at 24 h and 72 h of Sindbis treatment compared to control treatment.

To examine whether Sindbis viral vectors influence gene expression in hippocampal neurons, we prepared cultured organotypic slices of hippocampi from C57BL/6 mice. Half of the slices prepared from each mouse were injected with a buffered solution containing Sindbis viral vector coding eGFP and the other half with a control solution. Slices were transduced with the viral vector to have the majority (50–100%) of neurons express GFP. We isolated total RNA from the slices at 24 or 72 h after transduction. The integrity of isolated RNA was high and comparable between groups (average RIN: 9.1 ± 0.14). Copy RNA was synthesized and hybridized to a mouse gene expression microarray containing 44.000 features (Figure 1A). After hybridization, 43.020 features, encompassing 33.274 unique identifiers passed our detection criteria to be included in the gene expression analysis. The total change in gene expression as a consequence of Sindbis transduction was substantial: 27.5% of identifiers at 24 h and 19.1% at 72 h after transduction were significantly altered between transduced and control tissue. To gain insight into the major types of biological processes that were altered upon Sindbis transduction, we performed Gene Ontology (GO) over-representation and pathway analyses using Panther GO-Slim and Panther Pathways (Thomas et al., 2003; Mi et al., 2009). For this gene ontology analysis, we selected the transcripts that significantly changed by more than 2-fold, which represents 5,7% of identifiers at 24 h and 4.4% at 72 h after transduction (Figure 1B). A substantial number of gene expression alterations overlapped between 24 and 72 h conditions (Figure 1C). GO classes with an FDR-corrected *P*-value of < 0.05 were considered significant (Tables 1, 2). At both 24 and 72 h time points, the genes whose expression was most significantly altered between the Sindbis treated and control groups are strongly associated with gene groups involved in immunological processes (e.g., “Cytokine activity,” “Chemokine activity,” “Response to interferon-gamma”). These results suggest that massive exposure to Sindbis viral vectors evokes cytokine and chemokine mediated innate immune responses in hippocampal slice cultures.

**TABLE 1:** Gene Ontology analysis of genes that changed ≥2-fold significantly (FDR-corrected *p* < 0.05) upon 24 h of Sindbis-GFP treatment compared to control.

	**Pathways, Molecular Function and Biological Process identifiers that are enriched in the set of genes that are regulated by 24h of Sindbis transduction**						
**ID**	**Term**	**Annotated**	**Regulated**	**Expected**	**Fold Enrichment**	**Raw *P*-value**	**FDR**
**Pathways**							
P00031	Inflammation mediated by chemokine and cytokine signaling pathway	187	39	14.49	2.69	3.11E-07	2.52E-05
P00006	Apoptosis signaling pathway	80	21	6.2	3.39	8.24E-06	4.45E-04
P00054	Toll receptor signaling pathway	39	13	3.02	4.3	5.82E-05	2.36E-03
**Molecular function**							
GO:0003824	Catalytic activity	3107	331	240.82	1.37	1.70E-09	3.14E-07
GO:0005515	Protein binding	1855	199	143.78	1.38	6.86E-06	2.54E-04
GO:0005125	Cytokine activity	106	25	8.22	3.04	5.98E-06	2.77E-04
GO:0016491	Oxidoreductase activity	462	67	35.81	1.87	4.83E-06	2.98E-04
GO:0016787	Hydrolase activity	1350	152	104.64	1.45	9.88E-06	3.05E-04
GO:0008009	Chemokine activity	24	10	1.86	5.38	9.48E-05	2.51E-03
GO:0005102	Receptor binding	625	78	48.44	1.61	1.27E-04	2.95E-03
GO:0008233	Peptidase activity	306	44	23.72	1.86	3.20E-04	6.58E-03
GO:0005488	Binding	3686	337	285.7	1.18	8.97E-04	1.38E-02
GO:0005126	Cytokine receptor binding	55	13	4.26	3.05	9.83E-04	1.40E-02
GO:0005243	Gap junction channel activity	19	7	1.47	4.75	1.91E-03	2.53E-02
**Biological process**							
GO:0002376	Immune system process	525	88	40.69	2.16	4.02E-10	1.40E-08
GO:0034341	Response to interferon-gamma	44	20	3.41	5.86	1.08E-08	3.29E-07
GO:0065009	Regulation of molecular function	313	55	24.26	2.27	1.97E-07	5.33E-06
GO:0050790	Regulation of catalytic activity	263	46	20.38	2.26	3.22E-06	6.52E-05
GO:0019221	Cytokine-mediated signaling pathway	40	15	3.1	4.84	5.02E-06	9.38E-05
GO:0016032	Viral process	11	7	0.85	8.21	1.54E-04	2.67E-03
GO:0000165	MAPK cascade	240	37	18.6	1.99	2.38E-04	3.61E-03
GO:0006520	Cellular amino acid metabolic process	186	31	14.42	2.15	2.68E-04	3.84E-03
GO:0040011	Locomotion	248	37	19.22	1.92	4.49E-04	5.74E-03
GO:0006950	Response to stress	488	60	37.82	1.59	9.97E-04	1.15E-02
GO:0032502	Developmental process	1063	114	82.39	1.38	9.94E-04	1.21E-02
GO:0006629	Lipid metabolic process	361	47	27.98	1.68	1.27E-03	1.41E-02
GO:0009063	Cellular amino acid catabolic process	50	12	3.88	3.1	1.36E-03	1.44E-02
GO:0019220	Regulation of phosphate metabolic process	351	46	27.21	1.69	1.50E-03	1.52E-02
GO:0006955	Immune response	296	39	22.94	1.7	2.65E-03	2.47E-02
GO:0006968	Cellular defense response	84	16	6.51	2.46	2.56E-03	2.49E-02
GO:0006928	Cellular component movement	350	44	27.13	1.62	3.93E-03	3.41E-02
GO:0009605	Response to external stimulus	300	39	23.25	1.68	3.83E-03	3.44E-02
GO:0030097	Hemopoiesis	11	5	0.85	5.86	4.27E-03	3.58E-02
GO:0007399	Nervous system development	218	30	16.9	1.78	5.87E-03	4.60E-02
GO:0006796	Phosphate-containing compound metabolic process	1084	110	84.02	1.31	6.15E-03	4.67E-02

*Fisher’s exact test with FDR correction was performed. Annotated = number of genes on the array with a specific identifier. Regulated = number of genes in the list of regulated genes with a specific identifier. Expected = number of genes with a specific identifier that is expected to occur in the list of regulated genes, if the regulated genes were a random subset of the annotated genes.*

**TABLE 2:** Gene Ontology analysis of genes that changed ≥ 2-fold significantly (FDR-corrected p < 0.05) upon 72h of Sindbis-GFP treatment compared to control.

**Pathways, Molecular Function and Biological Process identifiers that are enriched in the set of genes that are regulated by 72h of Sindbis transduction**							
**ID**	**Term**	**Annotated**	**Regulated**	**Expected**	**Fold enrichment**	**Raw *P*-value**	**FDR**
**Pathways**							
P00031	Inflammation mediated by chemokine and cytokine signaling pathway	187	33	11.23	2.94	2.78E-07	2.25E-05
P00054	Toll receptor signaling pathway	39	11	2.34	4.7	8.83E-05	4.77E-03
P00006	Apoptosis signaling pathway	80	15	4.8	3.12	2.77E-04	1.12E-02
**Molecular function**							
GO:0003824	Catalytic activity	3107	258	186.58	1.38	4.02E-08	3.72E-06
GO:0016787	Hydrolase activity	1350	128	81.07	1.58	8.03E-07	3.72E-05
GO:0005515	Protein binding	1855	165	111.39	1.48	6.64E-07	4.09E-05
GO:0005125	Cytokine activity	106	21	6.37	3.3	8.72E-06	3.23E-04
GO:0005488	Binding	3686	280	221.35	1.26	1.69E-05	5.21E-04
GO:0008009	Chemokine activity	24	9	1.44	6.24	6.48E-05	1.50E-03
GO:0005102	Receptor binding	625	63	37.53	1.68	1.84E-04	3.78E-03
GO:0016788	Hydrolase activity, acting on ester bonds	388	43	23.3	1.85	2.89E-04	5.34E-03
GO:0005243	Gap junction channel activity	19	7	1.14	6.14	4.65E-04	7.82E-03
GO:0008233	Peptidase activity	306	35	18.38	1.9	7.73E-04	1.19E-02
GO:0016491	Oxidoreductase activity	462	47	27.74	1.69	8.71E-04	1.24E-02
GO:0005509	Calcium ion binding	144	20	8.65	2.31	1.06E-03	1.39E-02
GO:0000166	Nucleotide binding	115	16	6.91	2.32	3.46E-03	3.77E-02
GO:0005126	Cytokine receptor binding	55	10	3.3	3.03	3.39E-03	3.92E-02
**Biological process**							
GO:0002376	Immune system process	525	79	31.53	2.51	2.20E-12	1.78E-10
GO:0034341	Response to interferon-gamma	44	17	2.64	6.43	2.77E-08	1.35E-06
GO:0019221	Cytokine-mediated signaling pathway	40	12	2.4	5	2.55E-05	6.89E-04
GO:0016032	Viral process	11	7	0.66	10.6	3.35E-05	8.15E-04
GO:0006968	Cellular defense response	84	17	5.04	3.37	4.86E-05	1.07E-03
GO:0006950	Response to stress	488	54	29.3	1.84	5.88E-05	1.19E-03
GO:0006631	Fatty acid metabolic process	156	24	9.37	2.56	1.07E-04	2.01E-03
GO:0000165	MAPK cascade	240	31	14.41	2.15	1.73E-04	3.00E-03
GO:0050790	Regulation of catalytic activity	263	33	15.79	2.09	1.88E-04	3.05E-03
GO:0065009	Regulation of molecular f unction	313	37	18.8	1.97	2.43E-04	3.70E-03
GO:0016337	Cell-cell adhesion	115	18	6.91	2.61	5.08E-04	7.27E-03
GO:0032502	Developmental process	1063	92	63.83	1.44	7.28E-04	9.82E-03
GO:0006629	Lipid metabolic process	361	39	21.68	1.8	9.10E-04	1.11E-02
GO:0040011	Locomotion	248	30	14.89	2.01	8.75E-04	1.12E-02
GO:0006636	Unsaturated fatty acid biosynthetic process	5	4	0.3	13.32	1.02E-03	1.13E-02
GO:0009063	Cellular amino acid catabolic process	50	10	3	3.33	1.84E-03	1.87E-02
GO:0006955	Immune response	296	32	17.77	1.8	2.42E-03	2.18E-02
GO:0035556	Intracellular signal transduction	789	69	47.38	1.46	2.97E-03	2.58E-02
GO:0006633	Fatty acid biosynthetic process	57	10	3.42	2.92	4.25E-03	3.56E-02
GO:0007399	Nervous system development	218	25	13.09	1.91	4.51E-03	3.65E-02
GO:0006520	Cellular amino acid metabolic process	186	22	11.17	1.97	5.69E-03	4.46E-02

*Fisher’s exact test with FDR correction was performed. Annotated = number of genes on the array with a specific identifier. Regulated = number of genes in the list of regulated genes with a specific identifier. Expected = number of genes with a specific identifier that is expected to occur in the list of regulated genes, if the regulated genes were a random subset of the annotated genes.*

In addition, biological process categories that seem unrelated to post-mitotic neurons, such as “Cell proliferation,” “Locomotion” and “Macrophage activation,” were overrepresented when Sindbis treated cultured hippocampal slices were compared to control treated slices at either 24 or 72 h post-injection. In response to injury or pathogen invasion, quiescent ramified microglia proliferate and transform into reactive microglia (Kreutzberg, 1996; Stence et al., 2001). We specifically investigated the commonly used reactive microglia markers CD40 antigen, CD68 antigen, Cx3cr1, Icam1, and Tmem119 to see whether these were upregulated in Sindbis treated slices, as would be expected from microglial activation (Streit et al., 1989; Graeber and Streit, 1990; Slepko and Levi, 1996; Benveniste et al., 2001). Indeed, levels of CD40, CD68, and Icam1 were increased at both 24 and 72 h after transduction. Tmem119 levels were unaffected at 24 h, however, showed a significant increase at 72 h. Astrocyte markers such as Gfap, S100 beta, vimentin and Aldh1a1 that are associated with reactive astrocytes were decreased. These data suggest that a proportion of gene expression changes might be due to the activation of glial cells. Notably, genes classified in the “Apoptosis signaling pathway” were overrepresented at both 24 and 72 h time points post-Sindbis transduction (Tables 1, 2). This may indicate that the exposure to Sindbis vectors triggers apoptotic cell death in cultured slices. However, we cannot distinguish whether this involves apoptosis signaling in glia or neurons.

We also measured gene expression changes over time. The comparison of 24h and 72h of Sindbis transduction yielded 101 genes that were at least 2-fold up- or down-regulated (75 up and 26 down) (Figure 1B). This list corresponds to 0.3% of the total genes detected in the microarray, and shows over-representation of the Pathways identifier “Inflammation mediated by chemokine and cytokine signaling pathway” (Table 3), suggesting an evolving immune response between 24 and 72 h. The comparison of 24 and 72 h of control treatment yielded 32 genes (0.1% of the total amount of genes) that were at least 2-fold up- or down-regulated (29 up and 3 down) (Figure 1B), possibly reflecting maturation or aging of hippocampal cells in organotypic slices.

**TABLE 3:** Gene Ontology analysis of genes that changed > 2-fold significantly (FDR-corrected p < 0.05) between 24h and 72h of Sindbis-GFP treatment.

**Pathways, Molecular Function and Biological Process identifiers that are enriched in the set of genes that are regulated between 24 and 72h of Sindbis transduction**							
**ID**	**Term**	**Annotated**	**Regulated**	**Expected**	**Fold Enrichment**	**Raw *P*-value**	**FDR**
**Pathways**							
P00031	Inflammation mediated by chemokine and cytokine signaling pathway	187	6	0.86	6.95	2.55E-04	4.14E-02
**Biological Process**							
GO:0002376	Immune system process	525	11	2.43	4.54	3.01 E-05	7.32E-03

*Fisher’s exact test with FDR correction was performed.Annotated = number of genes on the array with a specific identifier. Regulated = number of genes in the list of regulated genes with a specific identifier. Expected = number of genes with a specific identifier that is expected to occur in the list of regulated genes, if the regulated genes were a random subset of the annotated genes.*

### Effects of Sindbis-GFP Transduction on the Proteome

**FIGURE 2:**
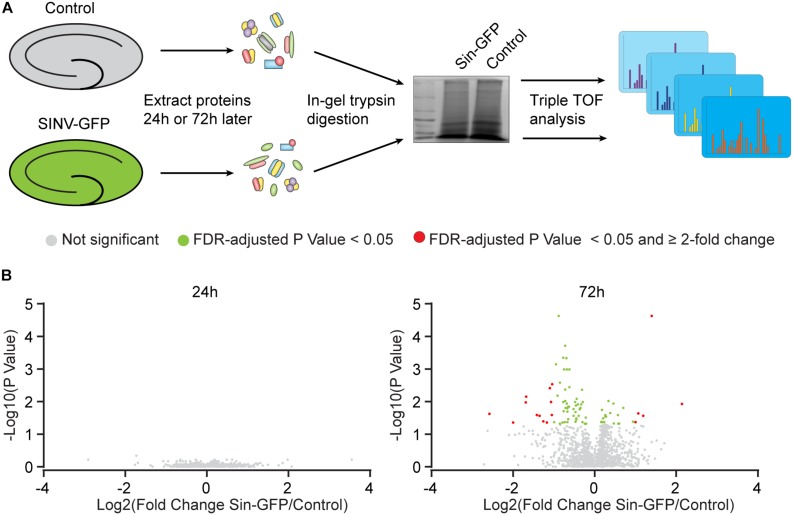
Protein expression changes caused by Sindbis transduction. **(A)** Schematic workflow (see methods): Organotypic hippocampal slices were injected with Sindbis-GFP (*n* = 6) or control treated (*n* =5), and analyzed 24 or 72 h after injection. **(B)** Comparisons in protein expression between Sindbis-GFP and control samples at 24h (left panel) and 72h (right panel). Volcano plots show fold change in gene expression (Log2) against Benjamini-Hochberg corrected *p*-value (–Log10) for individual proteins. Proteins that do not pass the significance threshold (*p* < 0.05) are shown in grey, those that change significantly by < 2-fold are shown in green, and those that change significantly by ≥2-fold are shown in red.

To establish insight into protein expression profiles that change as a consequence of Sindbis transduction, a proteomic analysis was performed. Organotypic slices of the mouse hippocampus were injected with Sindbis viral vector expressing eGFP (50–100% of cells GFP^+^) or control-treated, and at 24 or 72 h post-injection total protein fractions were isolated, in-gel trypsin digested and analyzed by liquid chromatography tandem mass spectrometry (LC-MS/MS) (Figure 2A). For identification of peptides originating from both mouse and Sindbis, the obtained data were searched against their respective FASTA files. In total 20.559 and 19.844 peptides were identified, encompassing 2.919 and 2.792 proteins in the 24 and 72 h dataset, respectively. As expected, GFP was detected in the Sindbis injected slices, but not in control slices. Since the viral vecors are replication deficient, Sindbis structural and non-structural polyproteins are not produced by Sindbis-transduced cells. However, these polyproteins remained detectable in organotypic slices up to 72 h post-injection (Table 4), indicating viral vector particles were still present in organotypic slices. Besides these Sindbis-related proteins, a set of immune-related proteins were only found expressed in slices injected with viral vectors (and therefore cannot be statistically compared), which were more numerous at 72 h than at 24 h (Table 4). These include proteins involved in virus detection and interferon induction (*DDX58, HA1L)*, key transcription factors activated by interferons (*STAT1/2*) and other interferon-stimulated proteins (*IFIT1/2/3*, *ISG15, ICAM1, GBP2, IIGP1*, and *IGTP*). Together, these protein level changes are reminiscent of an anti-viral innate immune response in brain tissue (Fensterl and Sen, 2014; Hidano et al., 2016; Miller et al., 2016).

**TABLE 4:** Proteins that were only detected in Sindbis-GFP treated slices (top), or only detected in control treated slices (middle), or changed ≥2-fold significantly (FDR-corrected *p* < 0.05) upon Sindbis-GFP treatment compared to control (bottom).

**Gene name**	**Protein name**	**24 h**	**72 h**
**Proteins detected only in all Sindbis treated samples**			
Poln	Sindbis non-structural polyprotein	✓	✓
Pols	Sindbis structural polyprotein	✓	✓
Gfp	Green fluorescent protein	✓	✓
Cep55	Isoform 2 of Centrosomal protein of 55 kDa	✓	
Ddx58	Probable ATP-dependent RNA helicase DDX58		✓
Gbp2	Guanylate-binding protein 1		✓
H2-D1	D(B) glycoprotein		✓
Icam1	Isoform 2 of Intercellular adhesion molecule 1		✓
Ifit1	Interferon-induced protein with tetratricopeptide repeats 1	✓	✓
Ifit3	Interferon-induced protein with tetratricopeptide repeats 3	✓	
I gtp	Protein Igtp		✓
ligpl	Interferon-gamma-inducible GTPase Ifgga1 protein		✓
Isgl5	G1p2 protein		✓
Mvp	Major vault protein		✓
Rnf213	E3 ubiquitin-protein ligase RNF213	✓	✓
Stat1	Signal transducer and activator of transcription		✓
Stat2	Signal transducer and activator of transcription		✓
**Proteins detected only in all control treated samples**			
Atp2b2	Calcium-transporting ATPase	✓	
Endod1	Endonuclease domain-containing 1 protein		✓
Ptprf	Receptor-type tyrosine-protein phosphatase F		✓
Sparcl1	SPARC-like protein 1	✓	✓
**Proteins that are changed ≥2-fold significantly**			
Fth1	Ferritin heavy chain		✓
Glul	Glutamine synthetase		✓
Plpp3	Phospholipid phosphatase 3		✓
Hspb6	Heat shock protein beta-6		✓
Hspb1	Heat shock protein beta-1		✓
Chl1	Isoform 2 of Neural cell adhesion molecule L1-like protein		✓
Ftl1	Ferritin		✓
Psme1	Proteasome activator complex subunit 1 (Fragment)		✓
Ctnna1	Catenin (Cadherin associated protein), alpha 1		✓
Nfasc	Neurofascin		✓
Llgl1	Lethal(2) giant larvae protein homolog 1		✓
Cspg5	Isoform 2 of Chondroitin sulfate proteoglycan 5		✓
Vcam1	Vascular cell adhesion protein 1		✓
Neo1	Neogenin		✓
Slc7a14	Probable cationic amino acid transporter		✓
Aldhla1	Retinal dehydrogenase 1		✓
Hist1h1e	Histone H1.4		✓

For statistical analysis, we proceeded with proteins that were detected in at least half of the samples per condition. At 24 h, out of 1.671 detectable proteins none showed significant regulation by Sindbis viral vector after FDR correction (FDR = 0.05) (Figure 2B). At 72 h, out of 1.619 proteins, 84 proteins showed significant regulation at an FDR of 0.05 (5.2% of total; 23 up and 61 down), of which 17 showed significant regulation by at least 2-fold (5 up and 12 down) (Figure 2B). A number of higher expressed proteins are known to be induced by viral infection or interferon signaling (*FRIL1 FRIH, VCAM1 and PSME1)* (Mulvey et al., 1996; Calabresi et al., 2001). In addition, a number of extracellular matrix and cell adhesion proteins (*CSPG5, NCHL1, CTNA1* and *NFASC*) were lower expressed (Hillenbrand et al., 1999; Drees et al., 2005; Liu et al., 2011; Jin et al., 2018) (Table 4). PANTHER analysis of this list of 84 proteins yielded no significant associations with any known GO or Pathways identifiers. These experiments reveal that changes in protein expression as a consequence of Sindbis transduction were modest compared with gene expression changes, and were predominantly related to anti-viral innate immune responses.

### Effects of Sindbis-GFP Transduction on the Electrophysiological Properties

**FIGURE 3:**
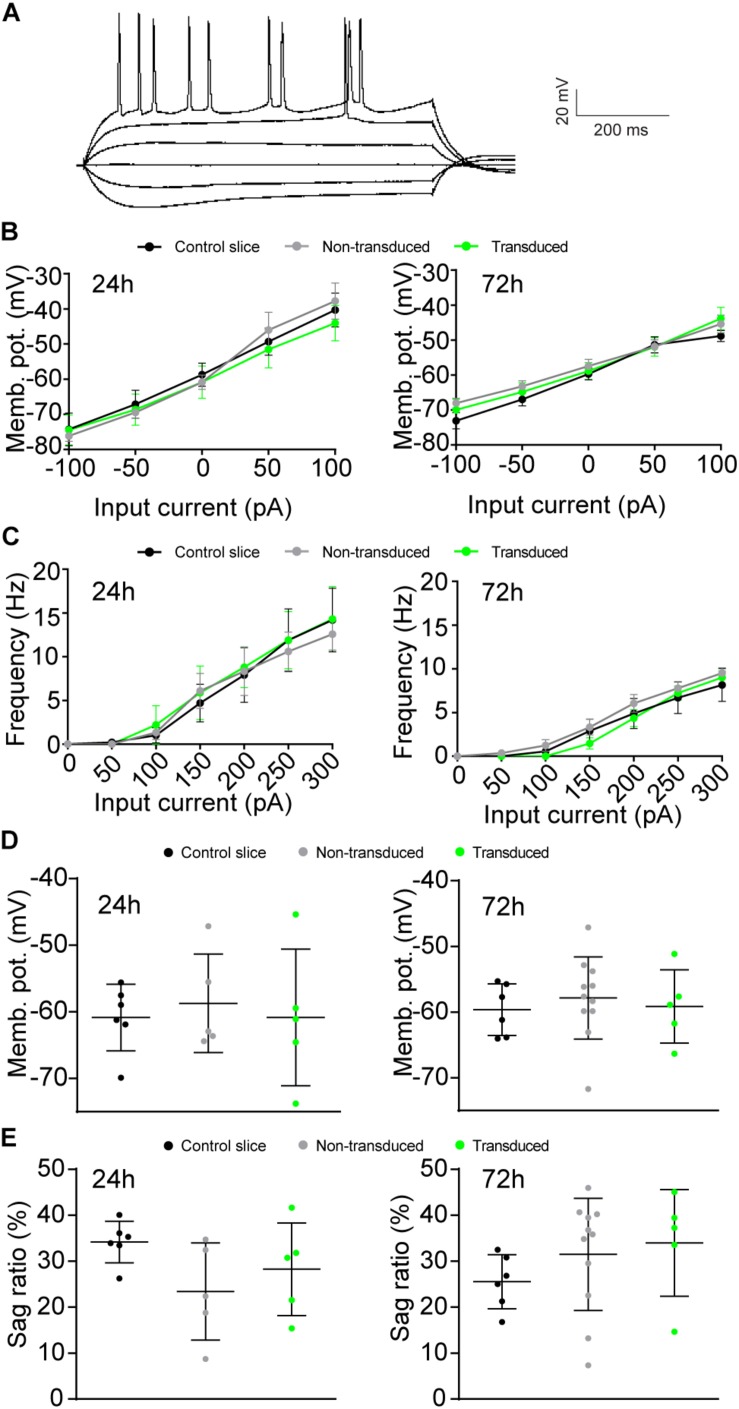
Effects of Sindbis transduction on neuronal excitability. **(A)** Example trace of whole-cell recordings. Recordings were made from Sindbis-transduced CA1 neurons (green; 24h: *n* = 5; 72 h: *n* = 5), neighboring non-transduced neurons (gray; 24 h: *n* = 5; 72 h: *n* = 11) and neurons from a control-treated slice (black; 24 h: *n* = 6; 72 h: *n* = 6). Membrane potential across current steps **(B)**, relation between frequency of APs and input current **(C)**, resting membrane potential **(D)**, and sag ratio **(E)** at both 24 and 72 h after injection are shown. Statistical test: Linear regression was used for comparison of slopes, error bars: SEM **(B,C)**; one-way ANOVA with Tukey’s multiple comparison test was used for comparison of the means, error bars: SD **(D,E)**.

We next assessed whether Sindbis transduction affected the electrophysiological properties of hippocampal neurons. We made comparisons between Sindbis-transduced CA1 pyramidal neurons and their neighboring non-transduced neurons in the same slice, and between neurons in slices from the same animal that were control treated. In these experiments we transduced organotypic hippocampal slices similarly as for the previous experiments (50–100% of neurons GFP^+^) and recorded at regions where ∼50–80% of neurons expressed GFP. Transduced and non-transduced CA1 neurons showed similar resting membrane potential and membrane potential changes across current injections at both the 24 and 72 h time points (Figures 3B,D). To assess whether Sindbis transduction affects the intrinsic excitability of CA1 neurons, we quantified the firing frequency per incremental current step to establish a frequency-current (F-I) relation. The F-I relation of GFP-expressing neurons was similar to that of non-transduced neurons at both 24h and 72h after injection (Figure 3C), indicating that Sindbis did not affect neuronal excitability. Hyperpolarization of CA1 neurons creates a voltage sag that is characteristic of HCN channel activation. GFP-expressing CA1 neurons did not show significant changes in sag ratio either at 24 or 72 h after Sindbis treatment (Figure 3E), indicating that Sindbis did not alter HCN currents.

**FIGURE 4:**
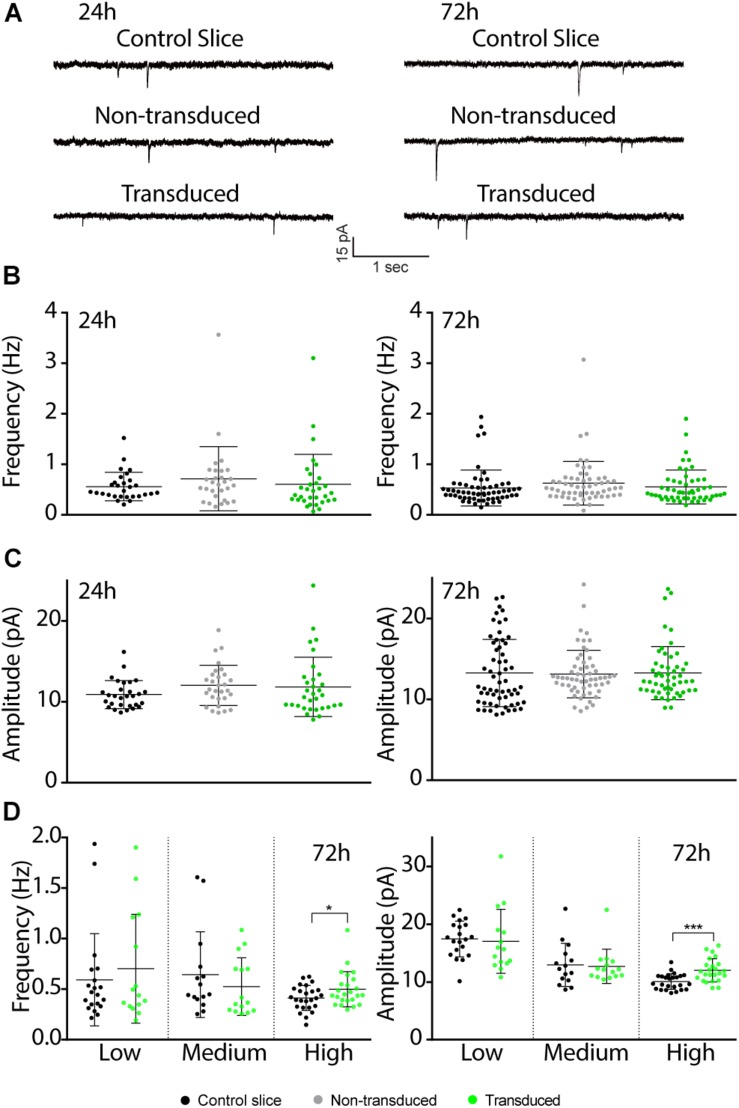
Effects of Sindbis transduction on synaptic currents. **(A)** Representative example traces of miniature EPSC recordings of Sindbis-transduced CA1 neurons, neighboring non-transduced CA1 neurons and CA1 neurons from a control-treated slice at 24 or 72 h post-injection. Average mEPSC frequency **(B)** and amplitude **(C)** of Sindbis-transduced neurons (green; *n*_24__*h*_ = 32, *n*_72h_ = 60), neighboring non-transduced neurons (gray; *n*_24__*h*_ = 29, *n*_72__*h*_ = 58) and neurons from a control-treated slice (black; *n*_24__*h*_ = 29, *n*_72__*h*_ = 54) at 24 and 72 h post-injection. Transduction rate at 24 h was 50–80% and at 72 h was 10–80%. **(D)** mEPSC frequency (left) and mEPSC amplitude (right) at 72 h after virus injection split between low (<20%), medium (20–50%) and high (50–80%) transduction rate from neurons in control-treated slices (black, *n*_*low*_ = 20, *n*_*medium*_ = 15, *n*_*high*_ = 25) and Sindbis-transduced neurons (green, *n*_*low*_ = 15, *n*_*medium*_ = 15, *n*_*high*_ = 24). Error bars: SD. Statistical test: Data normality was tested with Shapiro-Wilk normality test. Mean ranks were compared with Kruskal-Wallis test with Dunn’s multiple comparison (for comparison of three groups), or Mann-Whitney test (for comparison of two groups). ^*^p < 0.05, ^∗∗∗^*p* < 0.001.

Previous studies have shown that electrically evoked synaptic currents of CA1 neurons transduced with Sindbis-GFP are on average similar to those of neighboring non-transduced neurons 24–36 h after administration of the Sindbis viral vector (Hayashi et al., 2000; Kamenetz et al., 2003; Marie et al., 2005). To examine this further, we measured miniature excitatory postsynaptic currents (mEPSCs) at 24 or 72 h after Sindbis treatment. When we recorded GFP^+^ CA1 neurons 24h after transduction in regions containing 50–80% GFP^+^ neurons, the average amplitude and frequency of mEPSCs of Sindbis-transduced CA1 neurons were not significantly different from either neighboring non-transduced CA1 neurons, or neurons from a control treated slice (Figures 4A–C). For the 72 h time point, we tested the effect of Sindbis transduction in CA1 regions at three different transduction rates: low (<20%), medium (20–50%) and high (50–80%) levels of GFP+ CA1 neurons. For the data from three transduction rates combined, the mEPSC frequency and amplitude of transduced CA1 neurons are similar to those of control-treated neurons (Figures 4A–C). However, after splitting the data between the low, medium and high transduction rates, we did observe a significant increase in both amplitude and frequency in the high transduction rate group (Figure 4D) These results indicate that Sindbis transduction did not alter the number of active synapses, postsynaptic AMPA-receptor content or presynaptic glutamate release probability, except when neurons were recorded in regions of high (>50%) transduction rate and were recorded at 72 h after transduction.

## Discussion

In this study we analyzed the effects of Sindbis transduction of hippocampal neurons on the transcriptome, proteome and electrophysiological properties. At both 24 and 72 h after viral vector injection, substantial changes in gene expression were observed. The predominant changes in gene transcription relate to proteins involved in immunological responses, including the type-II interferon (IFN-γ), chemokine and cytokine pathways. At the protein level, the changes were also largely limited to proteins with a role in innate immune responses, although they occurred later (i.e., significantly altered at 72 h but not at 24 h), likely reflecting gene transcription preceding protein translation. The proteome changes were less pronounced compared with transcriptome changes: a large number of genes that showed a significant difference in gene expression upon Sindbis injection were not detected as a significant change on a protein level. This discrepancy may be explained by the notion that gene expression changes are not always accompanied by corresponding changes in protein levels due to post-transcriptional and post-translational regulation (De Sousa Abreu et al., 2009; Taylor et al., 2013).

In the CNS innate immune responses are predominantly mediated by glial cells, which can produce cytokines, interferons and chemokines upon exposure to viral particles (Miller et al., 2016). Our gene expression data demonstrate that glial cells were switched to an activated state when exposed to Sindbis particles. Possibly the changes in gene transcription and protein expression were in large part a consequence of a glia-mediated anti-viral innate immune response. A notable example of genes of which expression was significantly altered upon Sindbis injection without a detectable change in protein levels, are those involved in apoptotic signaling. Our data do not reveal whether these apoptotic genes were expressed in neurons or glial cells. We suspect these changes in apoptotic gene expression may be of glial origin, based on our observations that Sindbis causes an immune response and glial activation. This suspicion is supported by the lack of electrophysiological characteristics indicative of reduced health in GFP-expressing neurons. Possibly glia become activated after they are transduced by Sindbis viral vectors. However, previous studies show that Sindbis viral vectors have high selectivity for neurons over glial cell types (Ehrengruber et al., 2001). Alternatively, exposure of glia to viral particles may be sufficient to trigger a glial response, or transduced neurons may impact surrounding glial cells. We can also not exclude the possibility that effects we observed are due to immunogenicity of GFP as shown in recent years (Ansari et al., 2016).

Cytokine release upon the induction of an immune response can alter electrophysiological properties of neurons. For instance, exposure of neurons to interferons can lead to an increased excitability or to enhanced synaptic currents (Calvet and Gresser, 1979; Vikman et al., 2001; Strauss et al., 2004; Stadler et al., 2014). Similarly, long-term exposure of neurons to the glial tumor necrosis factor alpha (TNFα) promotes homeostatic scaling of synapse strength upon prolonged exposure (Beattie et al., 2002; Stellwagen and Malenka, 2006). Indeed, at high infection rates Sindbis infection did cause synaptic potentiation after prolonged periods of exposure to Sindbis particles. Notably, synaptic currents remained unaltered when applying low doses of viral particles obtaining fewer than 50% GFP^+^ neurons, possibly by reducing the strength of the anti-viral immune response. We therefore advocate using sparse (<50%) transduction for electrophysiological recordings on Sindbis- transduced neurons to minimize secondary effects on neuronal function due to glial activation and cytokine production. In addition, inclusion of control conditions such as recording neighboring non-transduced neurons and using control vectors is strongly recommended. We do not advocate using Sindbis as a gene transfer method to examine transcriptome changes in cell populations, as the transcriptome is widely affected upon transduction with Sindbis viral particles. However, it is conceivable that lower levels of Sindbis transduction may mitigate these gene expression changes. We conclude that, provided that proper control conditions are included, recombinant Sindbis is a suitable tool for studying short-term effects of transgene overexpression on neuronal and synaptic function.

## Data Availability

The datasets generated for this study can be found in ProteomeXchange Consortium via the PRIDE partner repository, PXD013634. The microarray datasets are available upon request.

## Author Contributions

SU, KB, AS, JV, and HK designed the experiments. SU, SvdS, NR, HX, KL, and HK performed the experiments and analyzed the data. SU and HK wrote the manuscript with input from all authors.

## Conflict of Interest Statement

The authors declare that the research was conducted in the absence of any commercial or financial relationships that could be construed as a potential conflict of interest.

## Footnotes

1 http://www.bioconductor.org
